# Comparative Metagenomic Analysis of Two Hot Springs From Ourense (Northwestern Spain) and Others Worldwide

**DOI:** 10.3389/fmicb.2021.769065

**Published:** 2021-11-26

**Authors:** María-Eugenia DeCastro, Juan-José Escuder-Rodríguez, Manuel Becerra, Esther Rodríguez-Belmonte, María-Isabel González-Siso

**Affiliations:** Grupo EXPRELA, Centro de Investigacións Científicas Avanzadas (CICA), Departamento de Bioloxía, Facultade de Ciencias, Universidade da Coruña, A Coruña, Spain

**Keywords:** thermophiles, extreme environments, hot springs, microbial communities, metagenomics, next generation sequencing

## Abstract

With their circumneutral pH and their moderate temperature (66 and 68°C, respectively), As Burgas and Muiño da Veiga are two important human-use hot springs, previously studied with traditional culture methods, but never explored with a metagenomic approach. In the present study, we have performed metagenomic sequence-based analyses to compare the taxonomic composition and functional potential of these hot springs. Proteobacteria, Deinococcus-Thermus, Firmicutes, Nitrospirae, and Aquificae are the dominant phyla in both geothermal springs, but there is a significant difference in the abundance of these phyla between As Burgas and Muiño da Veiga. Phylum Proteobacteria dominates As Burgas ecosystem while Aquificae is the most abundant phylum in Muiño da Veiga. Taxonomic and functional analyses reveal that the variability in water geochemistry might be shaping the differences in the microbial communities inhabiting these geothermal springs. The content in organic compounds of As Burgas water promotes the presence of heterotrophic populations of the genera *Acidovorax* and *Thermus*, whereas the sulfate-rich water of Muiño da Veiga favors the co-dominance of genera *Sulfurihydrogenibium* and *Thermodesulfovibrio*. Differences in ammonia concentration exert a selective pressure toward the growth of nitrogen-fixing bacteria such as *Thermodesulfovibrio* in Muiño da Veiga. Temperature and pH are two important factors shaping hot springs microbial communities as was determined by comparative analysis with other thermal springs.

## Introduction

Metagenomics has revolutionized microbial ecology, overcoming and complementing the traditional time-consuming culture methods. This approach has widely contributed to reveal the great microbial diversity of ecosystems previously thought to be lifeless and scarcely studied due to their irreproducible environmental conditions, such as hot springs. Nowadays, hot springs are considered ideal sites for studying the early life forms, or even extraterrestrial conditions (Konhauser et al., 2003; Pirajno, 2020). Furthermore, metabolically diverse microbial communities have been described in hot springs with very different physicochemical parameters (Sahay et al., 2017; Saxena et al., 2017), and some of their thermostable enzymes have been characterized as valuable biocatalysts with potential use in biotechnology and industry (Li and Liu, 2017; Ferrandi et al., 2018).

Previous studies have utilized comparative metagenomics to unveil the influence of physicochemical parameters in the diversity of thermophilic microbial communities inhabiting thermal springs (Menzel et al., 2015; Chiriac et al., 2017; Hussein et al., 2017; Sahoo et al., 2017; Mehetre et al., 2018). Among those, temperature and pH have been frequently identified as important factors shaping hot springs microbial populations (Chaudhuri et al., 2017; Mahato et al., 2019; Podar et al., 2020). Numerous investigations have reported a decrease in hot spring biodiversity with increasing temperature (Sharp et al., 2014; Li et al., 2015; Lavrentyeva et al., 2018). Chan et al. (2017) found that species richness and evenness in Malaysian hot springs were negatively correlated with temperature, and thus the composition of the microbial community was determined by this parameter. A significant role of temperature regulating the distribution of hot spring microbial communities was also reported in the Tibetan Plateau Geothermal Belt (China) (Guo et al., 2020) and in Odisha (India) (Badhai et al., 2015). Contrariwise, Power et al. (2018) studied 925 geothermal springs across New Zealand and determined that pH was the main factor influencing hot spring diversity at temperatures below 70°C, while temperature had a significant effect on the microbial distribution at those hot springs with water temperature above 70°C.

From all the thermal spots found across Ourense (Galicia, Northwestern Spain), As Burgas and Muiño da Veiga hot springs are located just 5 km away, but their waters show very different chemical and mineral composition due to the high geologic variability of the region (González-Barreiro et al., 2009; López et al., 2019). The diatom communities and the lipolytic enzyme-producing thermophiles inhabiting As Burgas have been previously investigated by traditional methods (Deive et al., 2013; Leira et al., 2017); nevertheless, the sequencing of the whole environmental DNA, known as shotgun metagenomics, has never been applied to study As Burgas or Muiño da Veiga microbiomes.

In this work, we have used metagenomics in conjunction with statistical tools to compare the microorganisms and community structure of As Burgas and Muiño da Veiga. Moreover, in an attempt to ascertain whether environmental conditions such as pH or temperature determine the microbial diversity and function of these nearby ecosystems, we have analyzed the functional and taxonomical profile of these geothermal springs and other geographically distant hot springs that encompass different pH and temperatures.

## Materials and Methods

### Sample Collection

**FIGURE 1:**
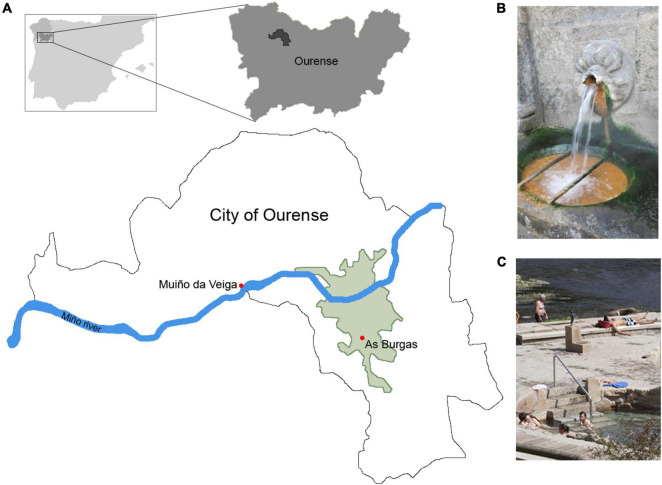
Details of As Burgas and Muiño Da Veiga hot springs. Geographical location **(A)**, As Burgas water spout **(B)**, and Muiño da Veiga water pump **(C)**.

As Burgas (BW) water, with temperature 66.3°C and pH 7.56 (González-Barreiro et al., 2009), was collected from As Burgas hot spring (GPS 42.334626, −7.865332), in Ourense (Galicia, Spain) (Figure 1A). Sampling was performed directly from the spout (Figure 1B) and not from a pool or reservoir exposed to light. The water sample was stored at room temperature and processed the next day when it was filtered through a nitrocellulose filter of 0.2 μm cutoff, using a bottle top filter holder (Nalgene) and a vacuum pump. Filter was stored at −20°C until metagenomic DNA extraction. Muiño da Veiga (MDV) water, with temperature 68°C and pH 7, was collected from a water pump (Figure 1C) located in Muiño da Veiga hot spring (GPS 42.352397, −7.909714), in Ourense (Galicia, Spain) (Figure 1A), following the same procedure described in As Burgas.

### DNA Extraction and Sequencing

Total DNA was isolated from the BW filters using the Metagenomic DNA Isolation Kit for Water (Epicentre Biotechnologies), according to the manufacturer’s protocol. Metagenomic DNA of both replicates was quantified using Qubit dsDNA HS Assay kit (Invitrogen) and prepared for Next-Generation Sequencing using the Accel-NGS^®^ 2S Plus DNA Library Kit (Swift Biosciences). The amplified libraries were checked with a Bioanalyzer 2100 (Agilent Technologies), and concentrations were quantified by Qubit dsDNA HS Assay kit (Invitrogen). Paired-end sequencing of the metagenomic DNA libraries was performed with 2 × 300 bp using the MiSeq sequencer (Illumina, San Diego, CA, United States) at San Diego State University. Similarly, metagenomic DNA from MDV was isolated from the filters using the Metagenomic DNA Isolation Kit for Water (Epicentre Biotechnologies), according to the manufacturer’s protocol. MDV metagenomic DNA was sequenced using the Illumina Hi-seq 1500 platform with 2 × 100 base read length by the sequencing services of Health in Code (A Coruña, Spain).

### Taxonomic Distribution and Statistical Analysis

Illumina reads were treated with PRINSEQ software (Schmieder et al., 2011) for quality control, removing all artificial duplicate reads and reads shorter than 60 base pairs. High-quality unassembled reads of both samples were uploaded into the Metagenomics Rapid Annotation using the Subsystem Technology (MG-RAST) v4.0.3 server (Meyer et al., 2008). BW and MDV metagenomes are publicly available under the accession numbers mgm4709018.3 and https://acortar.link/9uzzaS, respectively. MG-RAST server was used to assign the taxonomic profile of the metagenomic reads, with a maximum *e*-value of 1e^–05^, a minimum identity of 60%, and a minimum alignment length of 15 bp. The statistical analysis of the different taxonomic levels generated on MG-RAST was performed using the Statistical Analyses of Metagenomic Profiles (STAMP) (Parks et al., 2014) software. The significance of differences between proportions in the taxonomic distribution of BW and MDV samples was performed using the two-sided Fisher’s exact test, with the Newcombe–Wilson confidence interval method. Because *p*-values were not uniformly distributed, Benjamin–Hochberg false discovery rate was applied for correction. Results with *q* < 0.05 were considered significant. A difference of at least 1% and a twofold ratio between proportions was applied.

### Functional Analyses

Functional analyses were performed using the SEED subsystems annotation in the MG-RAST, with a maximum *e*-value of 1e^–05^, a minimum identity of 60%, and a minimum alignment length of 15 bp. The functional profiles generated by MG-RAST were statistically analyzed in the STAMP, with the same procedure and parameters described previously for the taxonomic analyses.

### Metagenome Sample Selection for Comparative Metagenomics

Apart from BW and MDV metagenomes, six other hot spring metagenomes were selected for the comparative analysis. To reduce the “type of sample” influences, this study included only hot spring water samples. All the samples selected are listed in Table 1 and publicly available on MG-RAST. The metagenomic sample of MDV is part of a present unpublished study from our group and was selected for its proximity to As Burgas hot spring, as both of them are located only a few kilometers away in the same Galician region (Ourense, Spain).

**TABLE 1:** Characteristics of the metagenomic data selected for this study.

Sample id	MG-RAST id	Hot spring	Location	pH	Temperature (°C)	Size (Mbp)	Sequencing method	References
BW	mgm4709018.3	As Burgas	Ourense, Spain	7.6	66	235.68	Illumina MiSeq	This study
MDV	https://acortar.link/9uzzaS	Muiño da Veiga	Ourense, Spain	7.0	68	10,408.06	Illumina Hi-seq 1500	Unpublished
Coamo	mgm4726046.3	Coamo	Coamo, Puerto Rico	8.2	47	33.10	Illumina MiSeq	Padilla-Del Valle et al., 2017
AT-4	mgm4555635.3	Atri	Odisha, India	7.4	58	22.6	Roche 454 GS-FLX	Badhai et al., 2015
HT-1	mgm4555636.3	Athamallik		7.4	56	11.96		
TB-3	mgm4555637.3	Tarabalo		7.3	57	22.79		
TP-2	mgm4555638.3	Taptapani		7.2	42	13.81		
Coquito	mgm4449206.3	El Coquito	Los Nevados, Colombia	2.7	29	53	Roche 454 GS-FLX Titanium	Jiménez et al., 2012
								

Taxonomic and functional profiles of the different samples were extracted from the MG-RAST. All of them are hot spring water samples that contain unassembled raw metagenomic reads in order to obtain information about the abundance of the sequences and, therefore, to be able to properly compare between samples. For each metagenome, sequence counts on MG-RAST were standardized against the total number of hits to remove bias in different sequencing efforts and library size.

## Results and Discussion

### DNA Extraction and Sequencing

After the quality control, 893,557 and 27,113,937 sequences with a total of 253,083,221 and 3,968,584,153 bp, an average length of 283 ± 71 and 146 ± 24 bp, and a GC content of 54 ± 12 and 44 ± 13% were uploaded to MG-RAST for BW and MDV, respectively. With this pipeline, 368,188 proteins were predicted for the BW sample and 194,410 were identified as protein features. For MDV, 6,422,118 proteins were predicted and 2,985,268 were identified as protein features.

### Comparative Analysis of Microbial Diversity Among the Hot Springs

**FIGURE 2:**
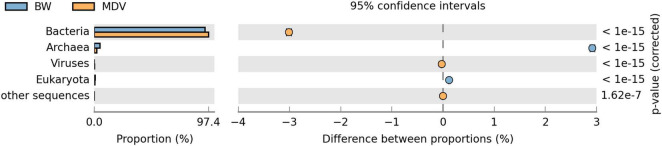
Comparative taxonomic profile of BW and MDV at domain level.

**FIGURE 3:**
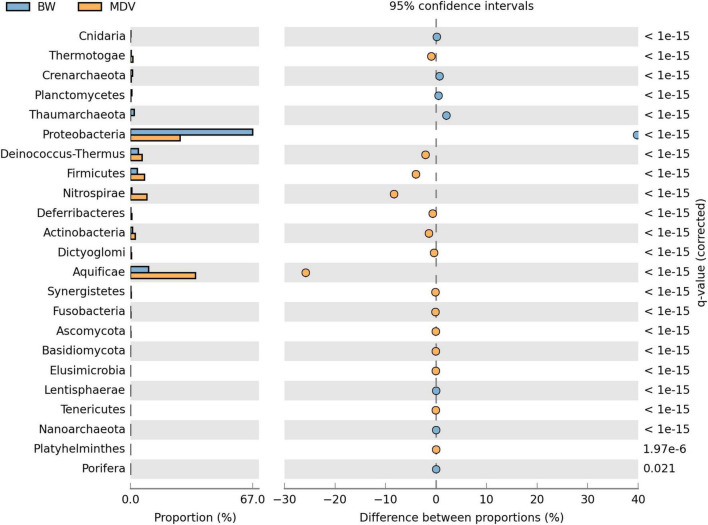
Comparative taxonomic profile of BW and MDV at phylum level. Only phylum with significant biological differences (*p* < 0.05, difference between proportions >1% and twofold of ratio between proportions, STAMP) were represented.

**FIGURE 4:**
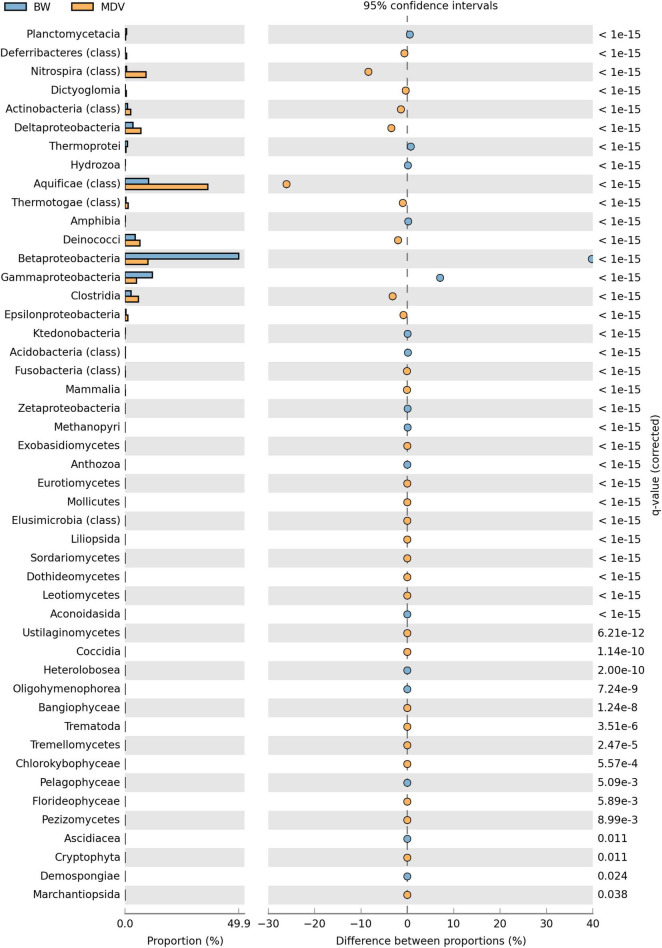
Comparative taxonomic profile of BW and MDV at class level. Only classes with significant biological differences (*p* < 0.05, difference between proportions >1% and twofold of ratio between proportions, STAMP) were represented.

At the domain level, the taxonomical profile generated by MG-RAST was similar in the BW and MDV samples. The highest representation of Bacterial sequences was found in both metagenomes (94.42 and 97.43%), followed by Archaea (4.88 and 1.96%), Eukaryota (0.66 and 0.54%), and Viruses (0.03 and 0.06%) (Figure 2). Proteobacteria (70.78 and 27.81%), Deinococcus-Thermus (4.54 and 6.55%), Firmicutes (3.91 and 7.89%), Nitrospirae (0.75 and 9.22%), and Aquificae (10.46 and 36.54%) were the more abundant phyla in the two samples (Figure 3). However, significant differences were found between both metagenomes, as Proteobacteria were predominant in BW while Aquificae, Firmicutes, and Nitrospirae were significantly more abundant in MDV. Classes Betaproteobacteria (42.8 and 9.88%) and Gammaproteobacteria (10.43 and 4.94%) were overrepresented in BW, in contrast with the prevalence of Aquificae (11.07 and 35.60%), Nitrospira (0.68 and 8.99%), and Deltaproteobacteria (3.43 and 6.84%) in MDV (Figure 4). A detailed list of the most abundant families found in both metagenomes can be found in Table 2.

**TABLE 2:** List and percentages of the four most abundant families within phyla Proteobacteria, Firmicutes, and Aquificae found in BW and MDV metagenomes.

	Phylum	Class	Order	Family	%
BW	Proteobacteria	Betaproteobacteria	Burkholderiales	Comamonadaceae	18.09
				Burkholderiaceae	5.13
			Rhodocyclales	Rhodocyclaceae	4.32
			Hydrogenophilales	Hydrogenophilaceae	4.12
	Firmicutes	Clostridia	Thermoanaerobacterales	Thermoanaerobacteraceae	0.76
			Clostridiales	Peptococcaceae	0.54
			Clostridiales	Clostridiaceae	0.52
		Bacilli	Bacillales	Bacillaceae	0.72
	Aquificae	Aquificae	Aquificales	Aquificaceae	10.37
				Hydrogenothermaceae	1.34
MDV	Proteobacteria	Betaproteobacteria	Burkholderiales	Comamonadaceae	3.65
				unclassified (derived from Burkholderiales)	1.59
				Burkholderiaceae	1.51
		Deltaproteobacteria	Desulfuromonadales	Geobacteraceae	2.39
	Firmicutes	Clostridia	Thermoanaerobacterales	Thermoanaerobacteraceae	1.46
			Clostridiales	Clostridiaceae	1.28
				Peptococcaceae	1.03
			Thermoanaerobacterales	Thermoanaerobacterales Family III. Incertae Sedis	0.97
	Aquificae	Aquificae	Aquificales	Aquificaceae	16.24
				Hydrogenothermaceae	20.30

Proteobacteria, Deinococcus-Thermus, Aquificae, and Firmicutes were also among the most abundant phyla found in the water of the Lobios hot spring (76°C, pH 8.2) located at 56.36 and 56.94 km in a straight line from As Burgas and Muiño da Veiga hot springs, respectively, in Ourense (Galicia) (López-López et al., 2015), and are frequently found in hot springs with different pH and temperatures (Huang et al., 2013; Chan et al., 2015; Paul et al., 2016).

The high relative abundance of phyla Proteobacteria and Firmicutes in the studied metagenomes is consistent with the results reported for other neutral springs, with different combinations of extreme environmental conditions, where these phyla were abundantly found (Filippidou et al., 2016; Chan et al., 2017). Nevertheless, the higher relative abundance of Firmicutes in MDV sample could be related to the temperature, similar to the results found in Bakreshwar (India) in which the hot spring with higher temperature showed more abundance of this phylum (Chaudhuri et al., 2017). A positive correlation between the abundance of phylum Firmicutes and temperature was also found in the hot springs of the Tibetan Plateau (Zhang et al., 2018).

Compared to BW, MDV water showed a higher abundance of phylum Nitrospirae (0.75 and 9.2%). Phylum Nitrospirae is composed mainly of aerobic chemolithotrophs, including microorganisms able to perform nitrification and sulfate-reducing bacteria (Garrity et al., 2001). Within phylum Nitrospirae, most species of the genus *Thermodesulfovibrio* have been isolated from thermal springs (Henry et al., 1994; Sonne-Hansen and Ahring, 1999; Haouari et al., 2008) and are characterized as obligately anaerobic thermophilic bacteria able to reduce sulfate and other sulfur compounds (Matsuura et al., 2016). *Thermodesulfovibrio* is the second most abundant genus in MDV while it is not abundant in BW (Table 3). This finding could be related to the relatively higher sulfate concentration of this hot spring compared to BW (López et al., 2019; Table 4). *Thermodesulfovibrio* was also abundantly found in several hot springs from Odisha (India), especially in AT-4 hot spring (Badhai et al., 2015) and in Borong hot spring (Najar et al., 2018).

**TABLE 3:** List of the 14 most abundant genera in BW and MDV metagenomes.

BW		MDV	
	% Sequences		% Sequences
***Acidovorax***	**6.89**	***Sulfurihydrogenibium***	**19.27**
***Thermus***	**4.50**	***Thermodesulfovibrio***	**8.78**
*Thiobacillus*	3.89	***Aquifex***	**6.60**
***Hydrogenobacter***	**3.58**	***Hydrogenobacter***	**4.92**
***Aquifex***	**3.56**	***Thermus***	**4.63**
*Polaromonas*	3.41	*Thermocrinis*	2.33
*Burkholderia*	2.39	*Geobacter*	2.33
*Nitrosopumilus*	2.26	***Acidovorax***	**1.34**
*Albidiferax*	1.67	*Meiothermus*	1.23
***Thermocrinis***	1.59	*Hydrogenobaculum*	1.15
***Nitrosomonas***	1.54	*Clostridium*	1.07
*Methylibium*	1.24	*Thiomonas*	0.90
*Verminephrobacter*	1.22	*Hydrogenivirga*	0.83
*Cupriavidus*	1.20	*Caldicellulosiruptor*	0.78

*Genera abundantly found in both metagenomes are highlighted in bold.*

*Percentages were generated by MG-RAST using data from the M5NR database.*

**TABLE 4:** Main physicochemical parameters of As Burgas and Muiño Da Veiga waters.

	*T* (°C)	*C* (uS/cm)	pH	Na^+^	K^+^	Ca^2+^	CO_3_H^–^	NH_4_^+^	S	SO_4_^2–^	NO_2_^–^	NO_3_^–^
Muiño Da Veiga	68	406	7	103	4	2	246	0.58	nd	9	0.1	0.5
As Burgas	66.3	960	7.56	102	8.2	11	0.69	0.94	0.02	3	nd	0.3

*Concentrations are reflected as mg ml^–1^.*

*Except for pH and temperature, all data were extracted from Delgado-Outeiriño et al. (2009) analyses.*

With 19.27% of sequences assigned, *Sulfurihydrogenibium* (phylum Aquificae) is the predominant genus in MDV (Table 3). Bacteria belonging to this genus are neutrophilic and thermophilic, microaerobic or facultatively anaerobic, chemolithoautotrophic, or facultatively heterotrophic microorganisms (O’Neill et al., 2008). *Sulfurihydrogenibium* is also among the 14 most abundant genera found in BW, and this finding is consistent with previous reports, as this genus has been frequently found to be the most prevalent in circumneutral hot springs with temperatures below 75°C (O’Neill et al., 2008). For example, in Kamchatka hot springs, it was found that lithoautotrophic bacteria from the genus *Sulfurihydrogenibium* were the most abundant in those springs with near-neutral pH (Merkel et al., 2017). Similar results were found in the analysis of six geothermal springs from Yellowstone National Park, in which *Sulfurihydrogenibium* sp. dominated in neutral sulfide-rich areas (Takacs-Vesbach et al., 2013).

On the contrary, dominance in BW is more distributed among different genera (Table 3). *Acidovorax* (phylum Proteobacteria), *Thermus* (phylum Deinococcus-Thermus), *Thiobacillus* (phylum Proteobacteria), and *Hydrogenobacter* (phylum Aquificae) are the most abundant genera in this ecosystem. *Acidovorax* members are aerobic and chemoorganotrophic, using oxygen as the terminal electron acceptor, or in some species using the heterotrophic denitrification of nitrate (Heylen et al., 2008). Although first described as mesophilic species, several studies across the world have now linked some Acidovorax species to moderate (Padilla-Del Valle et al., 2017; Saxena et al., 2017) to high temperature hot springs (Kozubal et al., 2012; Selvarajan et al., 2018), particularly to acid-sulfate springs or iron-rich springs.

*Thermus* species are generally thermophilic heterotrophs able to grow using different organic sources, while several can grow mixotrophically with inorganic electron donors (thiosulfate and elemental sulfur) for respiration (Skirnisdottir et al., 2001; Bjornsdottir et al., 2009; Murugapiran et al., 2013). Although most of them are aerobic bacteria, some members of this genus are facultative anaerobic, using NO^3–^, Fe^3+^, or S^0^ as terminal electron acceptors (Alvarez et al., 2014). Its respiratory flexibility suggests an important role of the genus *Thermus* in sulfur, metal, and nitrogen biochemical cycles in terrestrial geothermal springs (Zhou et al., 2020). At 9.66%, *Thermus* sp. is also abundant in MDV water. This finding agrees with the features described for this genus, with an optimum pH of 7.0–8.5 and an optimum temperature of 65–75°C (Massello et al., 2020); thus, *Thermus* sp. has been commonly found to be abundant in circumneutral thermal springs with temperatures between 60 and 80°C worldwide (Chan et al., 2017; Kaushal et al., 2018; Power et al., 2018).

The dominance of two heterotrophic genera such as *Acidovorax* and *Thermus* in BW metagenome might be related to the great variety of naturally occurring heteroatom-containing organic compounds reported in As Burgas water, mainly N- and O-containing compounds, some of them with aromatic ring(s) (González-Barreiro et al., 2009).

*Hydrogenobacter* sp., mainly constituted by chemolithoautotrophic hydrogen- and sulfur-oxidizing bacteria that use the reductive tricarboxylic acid cycle to fix CO_2_ (Aoshima et al., 2004; Sano et al., 2010), is the fourth most abundant genus in BW and MDV (Table 3). These results point to the members of genera *Hydrogenobacter* and *Sulfurihydrogenibium* as mainly responsible for carbon fixation in both ecosystems. The abundance of *Hydrogenobacter* sp. in BW and MDV could be attributed to pH, as this genus has been described as neutrophilic (Bonjour and Aragno, 1986) and has been predominantly found in neutral geothermal springs in which genus *Thermus* is frequently present (Massello et al., 2020). Abundance and co-occurrence of *Thermus* and *Hydrogenobacter* were previously reported for other hot springs, suggesting a possible metabolic association between both genera (Tomova et al., 2010; López-López et al., 2015; Ghilamicael et al., 2017; Bai and Peng, 2019).

Among the phylum Proteobacteria, members of the genus *Thiobacillus* are characterized by their diversity of metabolism, with a predominance of chemolitoautotrophy (Lavrentyeva et al., 2018). This genus includes sulfur-oxidizing species able to use elemental sulfur, sulfide, and thiosulfate as electron donors, such as *Thiobacillus ferrooxidans* and *T. thiooxidans*. Some species can use NO^3–^ as terminal electron acceptor like *Thiobacillus denitrificans* (Kumar et al., 2018); moreover, *T. ferrooxidans* has been described as potential nitrogen-fixing bacteria (Mackintosh, 1978; Rawlings and Kusano, 1994). Genus *Thiobacillus* is present with a 3.89% in BW, while it is not among the most abundant genera found in MDV. This genus was also detected in high abundance in Yumthang hot springs (India) (Panda et al., 2016), Shi-Huang-Ping acidic hot spring (Taiwan) (Lin et al., 2015), and Tsenkher Spring (Lavrentyeva et al., 2018).

Ammonia-oxidizing archaea (AOA) from the genus *Nitrosopumilus* (phylum Thaumarchaeota) were abundantly found in BW, while it is not among the 14 most abundant genera in MDV (Table 3). Several studies reported that Archaea rather than Bacteria are the main microorganisms driving ammonia oxidation in high-temperature hot springs environments (Chen et al., 2016). Although the ammonia concentration is considered to be an important factor influencing the community structure of ammonia oxidizers (Li et al., 2011; Hugoni et al., 2013; Chen et al., 2016) the dissimilarities in *Nitrosopumilus* abundance between both metagenomes could not be attributed to the differences in the ammonia concentration between BW and MDV waters (Table 4) as AOA have been reported to be able to grow with very low ammonia concentrations (Hatzenpichler, 2012).

**FIGURE 5:**
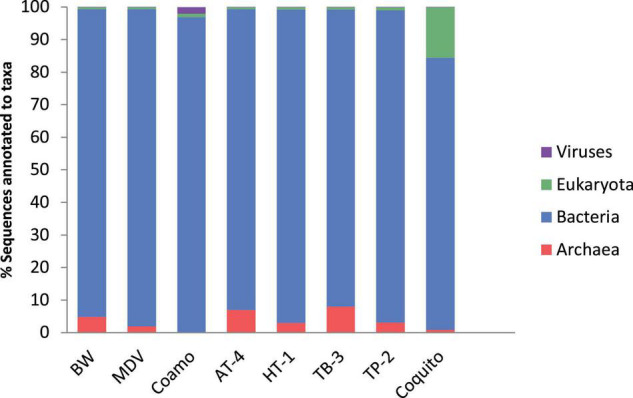
Comparative microbial diversity at different hot springs at domain level. Chart was generated using microbial abundance data. Each chart represents the percentage of abundance of each microbial group in a specific spring. Abundance values are generated from normalized data retrieved from MG-RAST.

When compared to the other studied hot springs, at the domain level, there is a predominance of Bacterial sequences followed by Archaea in the eight samples, except for El Coquito and Coamo, in which eukaryotic sequences and viruses are more abundant than archaeal sequences, respectively (Figure 5). The high abundance of Eukaryota in El Coquito metagenome might be related to its relatively low temperature (29°C) compared to the others. At this temperature, eukaryotic micro-algae can proliferate (Varshney et al., 2015) as important primary producers of the ecosystem taking advantage of the solar energy at the surface.

The high abundance of bacterial sequences in all the studied metagenomes, with temperatures ranging from 29 to 68°C, is comparable to other previously analyzed hot springs such as Lobios in Ourense (López-López et al., 2015), Comano in Italy (Pedron et al., 2019), Ma’in and Afra hot springs in Jordania (Hussein et al., 2017), or the hot springs from Bakreshwar (India) (Chaudhuri et al., 2017).

**FIGURE 6:**
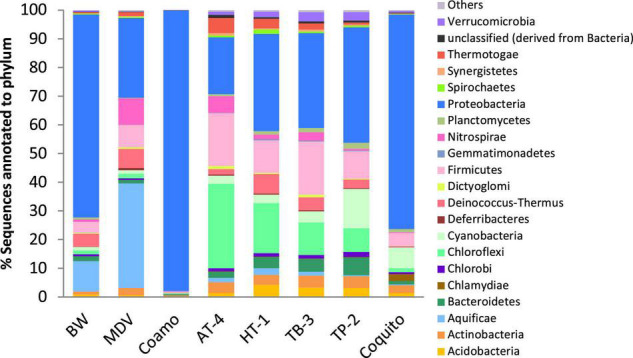
Comparative microbial diversity within Bacteria domain at phylum level. Others include those phyla with less than 0.3% sequences annotated in the eight metagenomes: Candidatus Poribacteria, Chrysiogenetes, Elusimicrobia, Fibrobacteres, Lentisphaerae, Tenericutes, and Fusobacteria.

Focusing on the Bacteria domain, at the phylum level, there is a clear predominance of Proteobacteria in BW, Coamo, HT-1, TB-3, TP-2, and Coquito metagenomes (Figure 6). The 95.03% of Proteobacterial sequences in Coamo hot spring metagenome is the highest of the eight metagenomes and could be a product of the library construction, as in the pCC1FOS system utilized by Padilla-Del Valle et al. (2017), *Escherichia coli* Epi300-T1*^R^* [F^–^ *mcr*A Δ(*mrr^–^hsd*RMS^–^*mcr*BC) φ80d*lac*ZΔM15 Δ*lac*X74 *rec*A1 *end*A1 *ara*D139 Δ(*ara*, *leu*)7697 *gal*U galK λ^–^ *rps*L *nup*G *trf*A *ton*A *dhfr*] was used as host, and the taxonomic assignment in MG-RAST shows that the more abundant sequences in Coamo thermal spring at the genus level are annotated as *Escherichia* (26%), a genus that is not frequently found as predominant in this kind of thermal environments. Moreover, although the study mentions the removal of vector pCC1FOS sequences, there is no evidence in the section “Materials and Methods” of the extraction of *E. coli* host sequences, which is an important step, as was reflected in other taxonomical studies that generated their sequences from a metagenomic library constructed in the pCC1FOS system (López-López et al., 2015; Rabelo-Fernandez et al., 2018; Soriano et al., 2018). On the contrary, this result might be a real reflection of Coamo microbial population, since this is a moderate temperature hot spring (47°C) and it has been demonstrated that several *E. coli* strains are thermotolerant bacteria that can evolve to grow at higher temperatures (Rudolph et al., 2010). Furthermore, *E. coli* has been detected in a thermal spring in Unkeswar (India) (Rekadwad and Khobragade, 2016) and in five hot springs of Eritrea (Ghilamicael et al., 2018). Additionally, in Coamo thermal spring, the genus *Microvirus*, constituted by single-stranded DNA viruses that infect Enterobacteria such as *Escherichia* (Krupovic and Forterre, 2011), is also abundantly found after taxonomic assignment by MG-RAST.

The high abundance of proteobacterial sequences in six of the eight studied metagenomes might be related to temperature, according to previous studies that have reported the predominance of phylum Proteobacteria in geographically distant moderate-temperature thermal springs such as Siloam in South Africa (Tekere et al., 2011), Attri and Yumthang in India (Panda et al., 2016; Tripathy et al., 2016), Ayer Hangat, Sungai Serai, and Dusun Tua in Malaysia (Chan et al., 2017), or Tatta Pani in Pakistan (Amin et al., 2017). Guo et al. (2020) suggested temperature as the main factor shaping the microbial community of 16 hot springs from the Tibetan Plateau as they found that hot springs with lower temperature (<45°C) showed a dominance of Proteobacteria and Nitrospirae, while in moderate- (55–70°C) to high-temperature (>70°C) geothermal springs, Aquificae, Deinococcus-Thermus, Thermodesulfobacteria, Thermotogae, and Cyanobacteria were the most abundant phyla. In agreement with Guo et al. (2020), from the eight hot springs studied here, proteobacterial sequences were the most abundant in those with lower temperatures (Coquito, Coamo, and TP-2) but, on the contrary, phylum Nitrospirae was not abundant. Moreover, the dominant phyla described for the moderate- to high-temperature Tibetan Plateau hot springs are relatively more abundant in the moderate- to high-temperature hot springs studied here, but Proteobacteria phylum is also abundant.

On the other hand, in all the Indian samples (AT-4, TP-2, HB-1, and TB-3), Firmicutes is a very abundant phylum with a percentage ranging from 9.7 to 18.6%, as reflected in Figure 5. Based on its abundance, Firmicutes is considered a signature phylum for circumneutral hot springs in several studies (Chan et al., 2017). Nevertheless, high abundance of Firmicutes has been reported in other Indian hot springs with alkaline pH and temperatures between 42 and 65°C such as Tuwa, Lasundra, Tulsi Shyam, and Bakreshwar (Ghelani et al., 2015; Mangrola et al., 2015a,b; Chaudhuri et al., 2017), suggesting that Firmicutes abundance in these samples might be due to other parameters related with the geographical proximity. Also, in all the Indian springs studied here, there is a clear abundance of Chloroflexi when compared with the rest of the samples. Bacteria from phylum Chloroflexi show a great variation in their metabolisms, with autotrophic, heterotrophic, and mixotrophic members (Bennett et al., 2020). The presence of Chloroflexi in the four Indian hot springs investigated by Badhai et al. (2015) and selected for this study is clearly correlated with temperature and with the distribution of phylum Cyanobacteria, as the abundance of phylum Chloroflexi increases with temperature while the abundance of cyanobacterial sequences decreases (Figure 6). Similarly, Wang et al. (2013) found that in Tibetan hot springs with moderate temperatures, abundances of phyla Cyanobacteria and Chloroflexi were negatively correlated. Another recent study also reported a decrease of the phototrophic Cyanobacteria with increasing temperature and maintenance of phototrophic Chloroflexi populations (Bennett et al., 2020).

Coquito and TP-2 are the hot springs with higher representation of Cyanobacteria in their microbial communities with 7.3 and 13.1% of cyanobacterial sequences, respectively. This finding agrees with their lower temperature (29°C in Coquito and 42°C in TP-2) that favors the survival of bacteria belonging to this phylum. Other studies have reported higher abundance of Cyanobacteria in lower-temperature hot springs (Sompong et al., 2005; Wang et al., 2013; Chan et al., 2017; Singh et al., 2018).

The presence of phylum Aquificae is higher in the two thermal springs from Ourense (Spain) with abundances of 10.43% in BW and 36.46% in MDV, in which it is the dominant phylum. This result might be related to their higher temperature, as some studies reported that the presence of Aquificae is positively correlated with temperature (Wang et al., 2013). Guo et al. (2020) also described higher abundance of phylum Aquificae in moderate- to high-temperature hot springs of the Tibetan Plateau than in those with lower temperatures. Other thermal springs with dominance of phylum Aquificae are Malangto (75.8°C, pH 5.08) and Balasbas (60.5°C, pH 5.20) in the Philippines (Huang et al., 2013).

Actinobacteria are present in all the studied metagenomes with percentages between 4.2% (TB-3) and 0.5% (Coamo). This phylum, first considered as characteristic from soil, nowadays has been reported as ubiquitous in hot springs as it has been found in several hot springs with very diverse pH and temperatures (Jiang et al., 2012; Valverde et al., 2012; Liu et al., 2016).

Phylum Deinococcus-Thermus is present in all the samples except for Coamo and Coquito metagenomes in which this phylum is almost absent. The absence of this phylum in Coquito and Coamo hot springs is related to their low temperatures, as all the members of the order Thermales require temperatures higher than 60°C to grow (Banerjee et al., 2014).

### Comparative Analysis of Functional Features Among the Hot Springs

**FIGURE 7:**
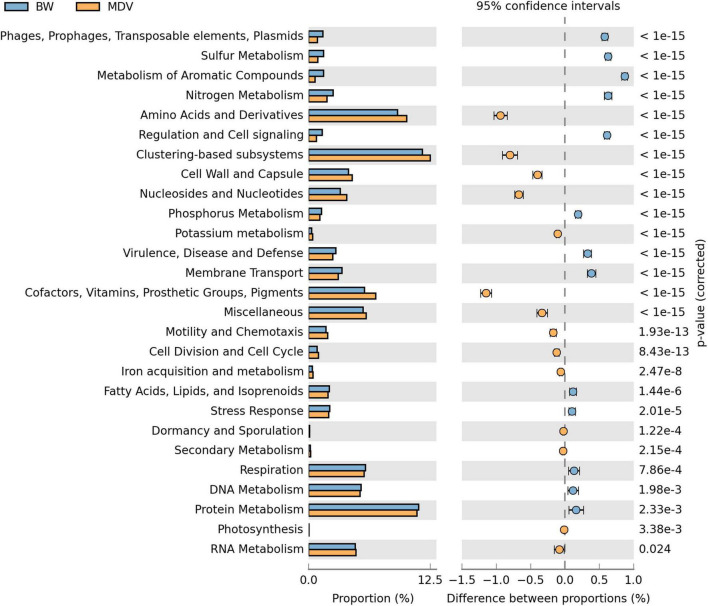
Comparative functional profile of BW and MDV at level 1 of the SEED subsystems. Only subsystems with significant biological differences (*p* < 0.05, difference between proportions >1% and twofold ratio between proportions, STAMP) were represented.

The functional profile generated with MG-RAST showed significant differences in proportion for subsystems at level 1 between BW and MDV metagenomes (considering *p* < 0.05 and differences of at least 1% and a twofold ratio between proportions) (Figure 7). Nevertheless, the clustering-based subsystems (11.68 and 12.48%), protein metabolism (11.29 and 11.12%), and amino acid and derivatives (9.12 and 10.06%) subsystems were the most abundant functional categories at level 1 in BW and MDV. The clustering-based subsystem groups protein families with functional coupling evidence but unknown function, and was also the most abundant subsystem found in the water of the Lobios hot spring in Ourense (López-López et al., 2015).

Interestingly, sulfur and nitrogen metabolism subsystems are overrepresented in BW. Focusing on these subsystems, there is a clear difference in the percentage of sequences assigned to each functional category at level 3 between both metagenomes (Table 5). In the sulfur metabolism, there is a predominance of sulfur oxidation affiliated sequences in BW with respect to MDV (Table 5). The relative abundance of sequences annotated as sulfur oxidation might be related to the presence of genera *Thermus*, *Thiobacillus*, *Thiomonas*, and *Sulfurihydrogenibium* in BW. Moreover, among the sequences annotated to the genera *Thermus*, *Thiobacillus*, and *Sulfurihydrogenibium*, reads associated to sulfur oxidation such as those coding for the SoxB and SoxY proteins were found in BW metagenome by functional annotation of the reads (Table 6).

**TABLE 5:** Percentages of sequences assigned to each functional category in sulfur metabolism, nitrogen metabolism, and CO_2_ fixation at level 3 in BW and MDV metagenomes.

		% Sequences	
		BW	MDV
Sulfur Metabolism	Inorganic Sulfur Assimilation	0.374	0.212
	Galactosylceramide and Sulfatide metabolism	0.002	0.009
	Release of Dimethyl Sulfide from Dimethylsulfoniopropionate	0	8.03e^–05^
	Sulfate reduction-associated complexes	0.156	0.164
	Sulfur oxidation	0.731	0.364
	Thioredoxin-disulfide reductase	0.100	0.112
	Alkanesulfonate assimilation	0.023	0.033
	Alkanesulfonates Utilization	0.002	0.004
	DMSP breakdown	2.742e^–04^	2.892e^–04^
	L-Cystine Uptake and Metabolism	0.003	0.007
	Taurine Utilization	0.003	0.004
	Utilization of glutathione as a sulfur source	0.018	0.016
Nitrogen Metabolism	Allantoin Utilization	0.013	0.013
	Amidase clustered with urea and nitrile hydratase functions	0	2.580e^–04^
	Ammonia assimilation	0.625	0.586
	Cyanate hydrolysis	0.012	0.011
	Denitrification	0.190	0.218
	Dissimilatory nitrite reductase	0.153	0.132
	Nitrate and nitrite ammonification	1.144	0.578
	Nitric oxide synthase	0.011	0.019
	Nitrilase	0	6.427e^–05^
	Nitrogen fixation	0.164	0.271
	Nitrosative stress	0.009	0.071
CO_2_ fixation	TCA cycle	0.487	0.311
	Calvin-Benson cycle	0.645	0.398
	Carboxysome	0.065	0.049
	CO_2_ uptake, carboxysome	0.295	0.163
	Photorespiration (oxidative C2 cycle)	0.266	0.297

**TABLE 6:** Functions involved in sulfur oxidation and DNRA associated to the genera *Thermus, Thiobacillus*, and *Sulfurihydrogenibium* found in As Burgas water metagenome.

		Number of sequences		
		*Thermus*	*Thiobacillus*	*Sulfurihydrogenibium*
Sulfur oxidation	SoxH protein, homolog		70	24
	Sulfide dehydrogenase [flavocytochrome C] flavoprotein chain precursor (EC 1.8.2.-)	13		
	Sulfite dehydrogenase cytochrome subunit SoxD	14		
	Sulfur oxidation molybdopterin C protein	12		
	Sulfur oxidation protein SoxB	23	201	10
	Sulfur oxidation protein SoxX	41		
	Sulfur oxidation protein SoxY	8	35	1
	Sulfur oxidation protein SoxZ	4	84	
	sulfur oxidation protein SoxA	23		
DNRA	Nitrate ABC transporter, ATP-binding protein		1	22
	Nitrate ABC transporter, permease protein		1	
	Nitrate/nitrite response regulator protein		97	
	Nitrate/nitrite sensor protein (EC 2.7.3.-)		102	
	Nitrate/nitrite transporter		9	
	Respiratory nitrate reductase alpha chain (EC 1.7.99.4)		49	
	Respiratory nitrate reductase beta chain (EC 1.7.99.4)		53	1
	Respiratory nitrate reductase delta chain (EC 1.7.99.4)		32	
	Respiratory nitrate reductase gamma chain (EC 1.7.99.4)		49	

The abundance of sequences related to nitrate and nitrite ammonification in BW, also known as dissimilatory nitrate reduction to ammonium (DNRA), could be associated with the predominance of genera *Thermus* and *Thiobacillus* in this metagenome, which can use NO^3–^ as the final electron acceptor, producing ammonia. In fact, some *Thiobacillus* sequences affiliated with genes involved in this pathway have been found (Table 6). Therefore, these genera might also be responsible for the relatively higher concentration of ammonia in BW when compared to MDV, stimulating the occurrence of ammonia oxidizers, such as those from the genus *Nitrosopumilus*, in the ecosystem.

The percentages of sequences assigned to denitrification, nitrogen fixation, and nitrosative stress functions are relatively higher in MDV than in BW (Table 5). Recent studies have suggested an association between nitrogen fixation and sulfate reduction in hot springs, proposing chemolithoautotrophic sulfate-reducing bacteria as mainly responsible for nitrogen fixation (Nishihara et al., 2018b). Moreover, all the genes necessary for nitrogen fixation have been found in several *Thermodesulfovibrio* species (Frank et al., 2016), and members of this genus have been pointed as possible nitrogen-fixing bacteria in Nakabusa hot springs in Japan (Nishihara et al., 2018a) and Mushroom spring in Yellowstone National Park (Thiel et al., 2017), although diazotrophic growth has not been demonstrated yet in the laboratory for any *Thermodesulfovibrio* species. In addition, the lower ammonia concentration of MDV (Table 3) might be promoting a selective pressure for the growth of diazotrophic bacteria able to fix N_2_, as has been generally reported in geothermal springs (Hamilton et al., 2014).

The elevated percentage of sequences involved in denitrification annotated in MDV could be associated with the dominance of genus *Sulfurihydrogenibium*, since the ability to denitrify completely to N_2_ has been described in several *Sulfurihydrogenibium* species (Takai et al., 2003). Indeed, by submitting the *Sulfurihydrogenibium* annotated reads to the Subsystems database in the MG-RAST, our data confirmed the presence of *Sulfurihydrogenibium* sequences involved in denitrification, such as Cytochrome cd1 nitrite reductase (EC:1.7.2.1) (Table 7).

**TABLE 7:** Reads from the genera *Sulfurihydrogenibium* annotated as denitrification functions in Muiño Da Veiga metagenome.

	*Sulfurihydrogenibium*
Cytochrome cd1 nitrite reductase (EC:1.7.2.1)	2464
Nitrous oxide reductase maturation protein, outer-membrane lipoprotein NosL	273
Nitrous oxide reductase maturation transmembrane protein NosY	440

These results suggest that the two main genera in MDV (*Sulfurihydrogenibium* and *Thermodesulfovibrio*) play an important role, not only in the sulfur cycle, but also in the nitrogen cycle. Moreover, the co-dominance of genera *Sulfurihydrogenibium* and *Thermodesulfovibrio* in MDV reveals the importance of sulfur metabolism in this hot spring and suggests the existence of a sulfur cycle in MDV geothermal spring between the two dominant genera, in which *Thermodesulfovibrio* would be reducing sulfate to sulfide through anaerobic sulfate respiration; sulfide is then abiotically or biotically oxidized to thiosulfate (Chen and Morris, 1972) and thiosulfate is used by *Sulfurihydrogenibium* as electron donor, producing sulfate (Takai et al., 2003). Furthermore, *Sulfurihydrogenibium* can grow autotrophically using elemental sulfur as an electron donor and nitrate as a final electron acceptor, producing sulfate, liberating N_2_, and coupling both sulfur and nitrogen cycles.

Focusing on the carbon fixation, an abundance of sequences affiliated to the Calvin–Benson Cycle is found in BW when compared to MDV. This might be related to the significantly higher proportion of Betaproteobacteria and Gammaproteobacteria in BW hot spring (Figure 4), as the autotrophic members of these taxonomic classes use the reductive pentose phosphate (Calvin-Benson) cycle to fix carbon (Hügler and Sievert, 2011). Sequences annotated within the tricarboxylic acid (TCA) cycle can be associated with the catabolism but also with the carbon fixation *via* the reductive TCA cycle performed by representatives of the genera *Hydrogenobacter*, *Thermocrinis*, and *Sulfurihydrogenibium*, among others (Hügler et al., 2007).

**FIGURE 8:**
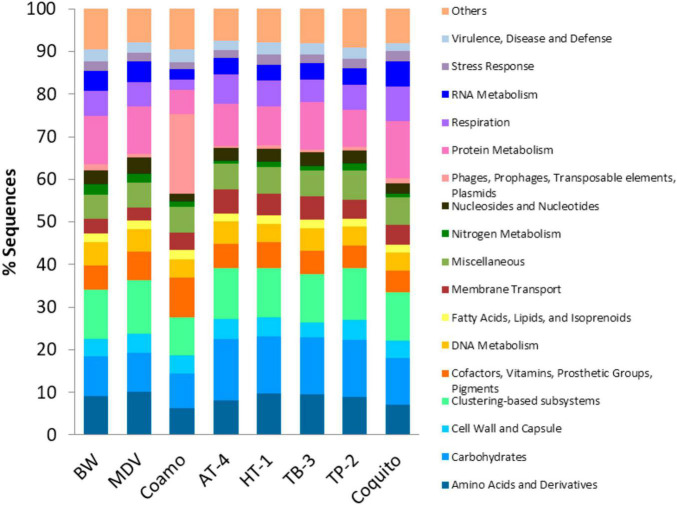
Comparative functional diversity at level 1. Others include those subsystems with less than 2% sequences annotated in the eight metagenomes: Cell division and cell cycle, dormancy and sporulation, iron acquisition and metabolism, metabolism of aromatic compounds, motility and chemotaxis, phosphorus metabolism, photosynthesis, potassium metabolism, regulation and cell signaling, secondary metabolism, and sulfur metabolism.

In relation to the functional profile of the studied metagenomes, the relative abundance of the 23 functions assigned by subsystems at level 1 is very similar in all the metagenomes with the exception of Coamo hot spring in which a higher proportion of sequences annotated as phages, prophages, transposable elements, and plasmids was detected (Figure 8). This result was expected due to the abundance of viral sequences (Figure 5) reported in Coamo metagenome compared to the other hot springs. On the contrary, sequences related to protein metabolism and cofactors, vitamins, prosthetic groups, and pigments were significantly lower in Coamo than in the rest of metagenomes.

## Conclusion

As the two hot springs from Ourense showed small differences in temperature and pH, the differences in bacterial community between BW and MDV are due to the previously described differences in the geochemical composition of their waters (González-Barreiro et al., 2009). The dominance of a heterotrophic population of the genus *Thermus* in BW is related to the high abundance of organic compounds detected in this geothermal spring (González-Barreiro et al., 2009), while in MDV, there is a predominance of chemolithoautotrophy performed by the genus *Sulfurihydrogenibium*.

Taxonomic and functional analyses showed that primary production in both hot springs is mainly driven by members of the genera *Sulfurihydrogenibium*, *Hydrogenobacter*, and *Thermocrinis*, which are sulfur and hydrogen oxidizers that can fix carbon using the reverse tricarboxylic acid pathway (rTCA). However, the differences in the abundance of these genera between the two metagenomes suggest that genera *Sulfurihydrogenibium* and *Hydrogenobacter* are mainly responsible for carbon fixation in MDV and BW, respectively.

The higher concentration of sulfate in MDV might be behind the abundance of the genus *Thermodesulfovibrio* in this hot spring in which the existence of a sulfur cycle between the two dominant genera (*Sulfurihydrogenibium* and *Thermodesulfovibrio*) is taking place.

In BW, ammonia oxidation driven by Archaea of the genus *Nitrosopumilus* can occur, while this genus is not abundant in MDV. On the other hand, the lower concentration of NH^4+^ in the MDV ecosystem could be driving a selection to nitrogen fixation, performed by members of the genus *Thermodesulfovibrio*, among others.

From a functional point of view, the results suggest a clear interaction between nitrogen and sulfur cycles in both metagenomes, as some members of the genera *Thermodesulfovibrio*, *Sulfurihydrogenibium*, and *Thermus* have been described as important players in both biogeochemical cycles and are abundantly found in BW and MDV hot springs, as well as the sequences related with metabolic pathways involved in nitrogen and sulfur cycles.

When compared to other geographically distant hot spring metagenomes, a clear effect of pH and temperature determining the taxonomy and function of hot springs microbial community can be detected. Phylum Cyanobacteria dominates in low-temperature hot springs, Proteobacteria is dominant in moderate-temperature hot springs, and Aquificae and Deninococcus-Thermus are more abundant in the high-temperature hot springs.

## Data Availability Statement

The datasets presented in this study can be found in online repositories. The names of the repository/repositories and accession number(s) can be found below: https://www.mg-rast.org, mgm4709018.3, https://www.mg-rast.org, https://acortar.link/9uzzaS.

## Author Contributions

M-ED did the writing, metagenomic DNA extraction, sequencing, and annotation of As Burgas water. J-JE-R did the metagenomic DNA extraction and annotation of Muiño da Veiga water. ER-B, MB, and M-IG-S supervised and reviewed the manuscript, providing comments and guidance during the work, and manuscript development. All authors contributed to the article and approved the submitted version.

## Conflict of Interest

The authors declare that the research was conducted in the absence of any commercial or financial relationships that could be construed as a potential conflict of interest.

## Publisher’s Note

All claims expressed in this article are solely those of the authors and do not necessarily represent those of their affiliated organizations, or those of the publisher, the editors and the reviewers. Any product that may be evaluated in this article, or claim that may be made by its manufacturer, is not guaranteed or endorsed by the publisher.
